# A commentary on ‘A dynamic nomogram for predicting intraoperative brain bulge during decompressive craniectomy in patients with traumatic brain injury: a retrospective study’

**DOI:** 10.1097/JS9.0000000000001348

**Published:** 2024-03-12

**Authors:** Dongzhou Zhuang, Shousen Wang

**Affiliations:** a Department of Neurosurgery, Fuzong Clinical Medical College of Fujian Medical University; b Fujian Provincial Clinical Medical Research Center for Minimally Invasive Diagnosis and Treatment of Neurovascular Diseases, Fuzhou, People’s Republic of China

*Dear Editor,*

Previous studies have explained the mechanism of uncontrolled brain swelling during surgery, and the venous system is considered to be an important factor^1^. Zhuang’s study suggests that the computed tomography value of the venous sinus is closely related to the occurrence of intraoperative brain bulge during decompressive craniectomy in patients with traumatic brain injury (TBI), suggesting that the venous system may be an important factor in intraoperative brain bulge (IOBB)^1^. In a rat model of subdural hematoma, the bridge vein is dragged, and the cortical vein is compressed, resulting in slow blood flow and even thrombosis. The effective diameter of the vein is reduced, and venous return is obstructed, which further exacerbates cerebral ischemia and edema and exacerbates the condition. When the hematoma was removed, the intracranial pressure was reduced, and the compression of brain tissue was relieved^2^. The blood flow in the intracranial artery and capillary area recovered quickly, while the venous blood flow decreased first and then recovered. There was a difference in reaction time between the arteries and veins, which led to instant congestion and blood stasis of the brain tissue, and the brain tissue quickly bulged in a short time. However, when venous compression occurs, brain swelling often occurs. What is the cause of brain swelling? Microcirculation disturbance secondary to trauma may be one of the key mechanisms explaining brain swelling.

The concept of cortical spreading depolarization (CSD) was proposed ~80 years ago. Previous studies have shown that CSD plays a role in the prognosis of patients with subarachnoid hemorrhage, ischemic stroke, or TBI^3–5^. Recent studies have shown that memantine inhibits cortical diffuse CSD and improves neurovascular function after traumatic brain injury^6^. These findings suggested that CSD plays an important role in the development of TBI. However, CSD has not been reported to be involved in the pathogenesis of brain swelling after TBI. Especially in TBI patients requiring craniotomy, uncontrolled brain swelling during surgery may also be associated with extensive neurovascular unit dysfunction caused by CSD. Therefore, an extensive neurovascular unit disorder caused by CSD may be a major mechanism for the occurrence of uncontrollable brain swelling during surgery in TBI patients.

After traumatic brain injury, intracranial hematoma and contusion cause intracranial hypertension, the blood flows through the arteriovenous system, and microcirculation is reduced. Moreover, the microvascular response can change to severe vasoconstriction and hypoperfusion (reverse coupling) due to the spontaneous burst of strong and recurrent CSD for several hours. Neuronal depolarization causes abnormal release of neurotransmitters, a large amount of glutamate promotes pericyte contraction and endothelial cell swelling, reduces capillary blood flow, and microcirculation thrombosis causes brain tissue hypoxia and energy metabolism disorders, which aggravate brain injury. When microcirculation is impaired to a certain extent, diffuse brain swelling may occur. After decompression surgery, arterial blood flow is the first to recover, and the first level of arterial blood is microcirculation. Extensive microcirculation disorders may prevent arterial blood from returning to the venous system or causing tissue space exudates, brain tissue congestion, and microcirculation disorders to lead to hypoxia and swelling of cells, resulting in uncontrollable brain swelling. If the microcirculation is successfully opened, the blood flows back to the second checkpoint, the vein. The obstruction of venous return can have the same effect. Only if the two levels are passed smoothly, and the cerebral blood flow is balanced in and out, is it possible to avoid uncontrollable brain swelling.

Therefore, extensive microcirculation disturbance and venous outflow obstruction may be two key points and arguably two important switches for the occurrence of uncontrolled brain swelling after trauma, especially during surgery. CSD may initiate microcirculation disturbance. CSD may be an important target for the treatment of posttraumatic brain swelling in the future, and further experiments are needed to verify this theory and develop more accurate and effective treatments.

## Ethical approval

Our study did not involve human participants or identifiable data; therefore, ethical committee approval was not required.

## Consent

Not applicable.

## Sources of funding

Joint Logistics Medical Key Specialty (Neurosurgery) Program (LQZD-SW).

## Author contribution

D.Z. and S.W. edited the letter.

## Conflicts of interest disclosure

The authors declare that they have no conflicts of interest.

## Research registration unique identifying number (UIN)

Not applicable.

## Guarantor

The submission is a correspondence. No new study was performed. All authors accept full responsibility for the submitted letter.

## Data availability statement

The correspondence is based exclusively on resources that are publicly available on the internet and duly cited in the ‘References’ section. No primary data were generated and reported in this manuscript. Therefore, the data has not become available in any academic repository.

## Provenance and peer review

Not applicable.

## References

[1] Zhuang D Li T Xie H . A dynamic nomogram for predicting intraoperative brain bulge during decompressive craniectomy in patients with traumatic brain injury: a retrospective study. Int J Surg, 110:909–920. 38181195 10.1097/JS9.0000000000000892 PMC10871569

[2] Wang C Xian L Chen X . Visualization of cortical cerebral blood flow dynamics during craniotomy in acute subdural hematoma using laser speckle imaging in a rat model. Brain Res 2020;1742:146901. 32445715 10.1016/j.brainres.2020.146901

[3] Owen B Vangala A Fritch C . Cerebral autoregulation correlation with outcomes and spreading depolarization in aneurysmal subarachnoid hemorrhage. Stroke 2022;53:1975–1983. 35196873 10.1161/STROKEAHA.121.037184 PMC9133018

[4] Binder NF Glück C Middleham W . Vascular response to spreading depolarization predicts stroke outcome. Stroke 2022;53:1386–1395. 35240860 10.1161/STROKEAHA.121.038085 PMC10510800

[5] Hartings JA Bullock MR Okonkwo DO . Spreading depolarisations and outcome after traumatic brain injury: a prospective observational study. Lancet Neurol 2011;10:1058–1064. 22056157 10.1016/S1474-4422(11)70243-5

[6] MacLean MA Muradov JH Greene R . Memantine inhibits cortical spreading depolarization and improves neurovascular function following repetitive traumatic brain injury. Sci Adv 2023;9:eadj2417. 38091390 10.1126/sciadv.adj2417 PMC10848720

